# A hypothesis on the biological origins and social evolution of music and dance

**DOI:** 10.3389/fnins.2015.00030

**Published:** 2015-02-18

**Authors:** Tianyan Wang

**Affiliations:** ^1^School of Life Science, Tsinghua UniversityBeijing, China; ^2^Ocean Science and Technology Division, Graduate School at Shenzhen, Tsinghua UniversityShenzhen, China; ^3^Gene and Cell Engineering Laboratory, Shenzhen Institutes of Advanced Technology, Chinese Academy of SciencesShenzhen, China

**Keywords:** music, dance, speech, emotion, origin, evolution, entrainment, Doppler effect

## Abstract

The origins of music and musical emotions is still an enigma, here I propose a comprehensive hypothesis on the origins and evolution of music, dance, and speech from a biological and sociological perspective. I suggest that every pitch interval between neighboring notes in music represents corresponding movement pattern through interpreting the Doppler effect of sound, which not only provides a possible explanation for the transposition invariance of music, but also integrates music and dance into a common form—rhythmic movements. Accordingly, investigating the origins of music poses the question: why do humans appreciate rhythmic movements? I suggest that human appreciation of rhythmic movements and rhythmic events developed from the natural selection of organisms adapting to the internal and external rhythmic environments. The perception and production of, as well as synchronization with external and internal rhythms are so vital for an organism's survival and reproduction, that animals have a rhythm-related reward and emotion (RRRE) system. The RRRE system enables the appreciation of rhythmic movements and events, and is integral to the origination of music, dance and speech. The first type of rewards and emotions (rhythm-related rewards and emotions, RRREs) are evoked by music and dance, and have biological and social functions, which in turn, promote the evolution of music, dance and speech. These functions also evoke a second type of rewards and emotions, which I name society-related rewards and emotions (SRREs). The neural circuits of RRREs and SRREs develop in species formation and personal growth, with congenital and acquired characteristics, respectively, namely music is the combination of nature and culture. This hypothesis provides probable selection pressures and outlines the evolution of music, dance, and speech. The links between the Doppler effect and the RRREs and SRREs can be empirically tested, making the current hypothesis scientifically concrete.

## Introduction

Music exists ubiquitously across human history and human culture (D'errico et al., 2003; Conard et al., 2009), and has the ability to evoke rewards, and other positive and negative emotions (Blood et al., 1999; Blood and Zatorre, 2001; Menon and Levitin, 2005; Koelsch et al., 2006; Wieczorkowska et al., 2006; Koelsch, 2014). However, the origins of music and musical emotions is still largely an enigma (Brown et al., 2000; Schyff, 2014).

Since Darwin (1871), more and more scientists believe that human music must be a biological adaptation (Wallin et al., 2001; Mithen, 2009). Studies on twins (Drayna, 2001) and congenital amusia (Peretz et al., 2007; Tan et al., 2014) indicated that pitch recognition provides a hereditary basis for musical ability. However, the critical question we need to answer is what selection pressures are responsible for the origins of music? Charles Darwin proposed that, like bird song and dance, human music and dance were promoted by sexual selection (Darwin, 1871). Sexual selection, considered as a selection pressure, has been acknowledged and developed by other scientists (Miller, 2000). Our life begins with a lullaby, matures with a wedding march and ends in funeral music. The social functions of music are so important, that many scientists argue that music originated and developed from social activities: strengthening the mother-baby connection (Dissanayake, 2000; Trehub, 2003) and social cohesion (Brown, 2000a; Freeman, 2000; Mithen, 2007). And there are also many hypotheses on the origins of musical emotions (Wallin et al., 2001; Brattico et al., 2009; Perlovsky, 2010; Altenmüller et al., 2013; Juslin, 2013; Patel and Iversen, 2014). All of these theories and hypotheses are reasonable to explain some aspects of music and musical emotions and will be significant in guiding future research, however, there are also more or less deficits (Hagen and Bryant, 2003). What's still under debate is which selection pressures are responsible for the origins and evolution of music and musical emotions.

Music is never alone, it is often accompanied by dance and other synchronized movements not only in humans (Repp, 2005), but also in other animals (Patel et al., 2009; Schachner et al., 2009; Cook et al., 2013). Music perception is also closely related to movement (Phillips-Silver and Trainor, 2005; Zatorre et al., 2007; Trainor et al., 2009; Maes et al., 2014). Besides dance and movement, music shares some common characteristics and neural circuits with speech (Falk, 2000; Marler, 2000; Zatorre et al., 2002; Patel, 2003; Koelsch et al., 2005; Masataka, 2009). And there is debate on the evolutionary relationship between music and speech (Bickerton, 2000; Molino, 2000). There are four main possibilities: music and speech evolved independently, both of them evolved from a common ancestor, music evolved from speech or speech evolved from music (Molino, 2000; Mithen, 2005; Besson et al., 2011).

If music appreciation is an evolutionary adaptation, is there any significant selection pressure for music and musical emotions? Is there any profound relation among music, dance and speech in the process of species evolution? Here I propose a hypothesis on the origins and evolution of music, dance, and speech from an biological and sociological perspective. Firstly, I integrate music and dance into a common form—rhythmic movements—through the Doppler effect of sound. Secondly, I suggest that the adaptation of organisms to external and internal rhythms is what has driven the formation of rhythm-related reward and emotion (RRRE) system, which enables animals to appreciate, search for and produce rhythmic movements and events. In part three and four, I elaborate on the origins and evolution of music, dance, and speech in animal and human society. In the last part, I use this hypothesis to reasonably explain results from previous studies, and give some directions on interesting avenues for future research.

## Rhythmic movements are the basic elements of music and dance

Cookery and romance have no direct connection with human evolution. However, their basic elements, food and sex are essential for survival and reproduction, respectively, which are integral to evolution. Similarly, if there is a significant biological basis for music and dance, the first question we need to answer is: what are the basic elements of music and dance? Although there are several elements such as, rhythm, melody, pitch, harmony, timbre, and dynamics in a solo (Jones et al., 2010), only pitch and rhythm are the basic ones (Platel et al., 1997; Krumhansl, 2000; Trainor and Unrau, 2012), which could alter emotional, behavioral, and physiological states in human beings (Schellenberg et al., 2000) and some kinds of animals (Snowdon and Teie, 2013). It seems that melody (the combination of rhythm and pitch) could be defined as the prototypical music, which has the ability to evoke rewards and emotions.

Melody is a series of rhythmic pitches in a time line (Cariani and Micheyl, 2012). In classical physics, the Doppler effect of sound indicates that the relative velocity between a sound source and the observer would influence the observed frequency *f* compare to the emitted frequency *f*_0_ in a stationary medium (Rosen and Gothard, 2009). The relationships between *f* and *f*_0_ are given by the formulas in Table 1. These formulas also indicated that a certain ratio of frequency (*f* / *f*_0_) denotes a certain relationship between *v*_*s*_ (the velocity of the source relative to the medium) and *v*_*r*_(the velocity of the receiver relative to the medium). I suggest that animals detect and calculate the source's movement pattern through their own movement pattern and the frequency ratio of sound. This ability may be valuable for the survival and reproduction, for example, some bats, whales and dolphins exploit the Doppler effect in echolocation for navigating and hunting (Nelson and Maciver, 2006; Au and Simmons, 2007; Ulanovsky and Moss, 2008; Parker et al., 2013; Corcoran and Conner, 2014). I suggest the human brain processes pitch intervals in music into movements by the interpreting of Doppler effect of sound, namely interpreting every pitch interval into a corresponding velocity of an abstract sounding event.

**Table 1:** **The relationships between observed frequency *f* and emitted frequency *f*_0_ in the Doppler effect (Rosen and Gothard, 2009)**.

	**Observed frequency**	**Receiver**	**Source**
Situation A	*f* = *f*_0_ *c* / (*c* + *v*_*s*_)	stationary	*v*_*s*_ = *c* (*f*_0_ − *f*) / *f*
Situation B	*f* = *f*_0_ (*c* + *v*_*r*_) / *c*	*v*_*r*_ = *c* (*f* − *f*_0_) / *f*_0_	stationary
Situation C	*f* = *f*_0_ (*c* + *v*_*r*_) / (*c* + *v*_*s*_)	moving (*v*_*r*_)	moving (*v*_*s*_)

c is the velocity of waves in the medium; v_*r*_ is the velocity of the receiver relative to the medium, which is positive or negative if the receiver is moving toward or moving away from the source, respectively; v_*s*_ is the velocity of the source relative to the medium which is positive or negative if the source is moving away from or moving toward the receiver, respectively.

The frequency ratio of every pitch interval in music is determined by equal temperament, which was first presented in high precision by the Chinese prince Zhu Zaiyu (Chinese: 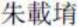) in 1584 (Kuttner, 1975). Therefore, rhythmic pitch intervals (the temporal sequence of frequency ratio between neighboring notes) in a melody represent rhythmic movements (Table 2). The rhythmic moving subject here is neither the instrument, nor the performer, but an abstract sounding event in our unconscious mind.

**Table 2:** **Invariant pitch interval in music represents invariant movement interpreted by the Doppler effect**.

**Pitch interval**	**Ratio of frequency**	**Velocity of ASE**	**Velocity of ASE in air (m/s)**
	*f* / *f*_0_	*c* (*f*_0_ / *f* − 1)	340 (*f*_0_ / *f* − 1)
−12	0.50000000	1.000 *c*	340.00
−11	0.52973155	0.888 *c*	301.83
−10	0.56123102	0.782 *c*	265.81
−9	0.59460356	0.682 *c*	231.81
−8	0.62996052	0.587 *c*	199.72
−7	0.66741993	0.498 *c*	169.42
−6	0.70710678	0.414 *c*	140.83
−5	0.74915354	0.335 *c*	113.85
−4	0.79370053	0.260 *c*	88.37
−3	0.84089642	0.189 *c*	64.33
−2	0.89089872	0.122 *c*	41.64
−1	0.94387431	0.059 *c*	20.22
0	1.00000000	0	0.00
1	1.05946309	−0.056 *c*	−19.08
2	1.12246205	−0.109 *c*	−37.09
3	1.18920712	−0.159 *c*	−54.10
4	1.25992105	−0.206 *c*	−70.14
5	1.33483985	−0.251 *c*	−85.29
6	1.41421356	−0.293 *c*	−99.58
7	1.49830708	−0.333 *c*	−113.08
8	1.58740105	−0.370 *c*	−125.81
9	1.68179283	−0.405 *c*	−137.83
10	1.78179744	−0.439 *c*	−149.18
11	1.88774863	−0.470 *c*	−159.89
12	2.00000000	−0.500 *c*	−170.00

ASE, the abstract sounding events in our unconscious mind.

This hypothesis could explain an unsolved problem in music perception, transposition invariance—with the correct pitch intervals between the notes, people could recognize a familiar tune irrespective of the absolute pitch of the beginning note (McDermott and Oxenham, 2008; Cariani and Micheyl, 2012; Trainor and Unrau, 2012). The reason may be that the invariant pitch intervals are representative of an invariant movement pattern.

**Figure 1:**
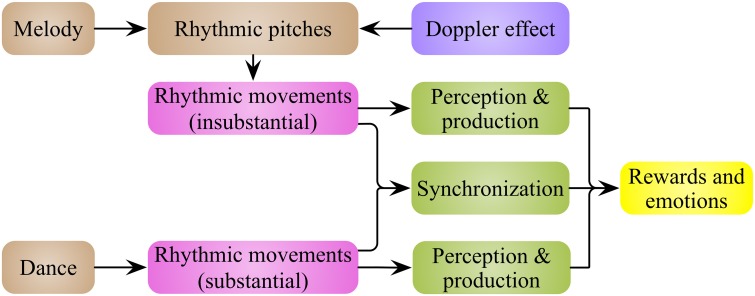
**Rhythmic movements are the basic elements of music and dance**. Music and dance are a series of rhythmic pitches and movements in time line, respectively. Based on Doppler effect of sound, rhythmic pitch intervals in music represent the rhythmic movements of an abstract event. It means that music and dance could be integrated into a common form**—**rhythmic movements. Rewards and emotions are evoked by the production, perception and synchronization of the rhythmic movements.

Melody and dance are temporal sequences of pitches and movements, respectively. As such, melody and dance can be integrated into a common form**—**rhythmic movements (Figure 1), with the perceiving, producing and synchronizing with these rhythmic movements inducing reward feelings, and other positive and negative emotions. The next question is: why and how rhythmic movements induce this emotional arousal?

## The generation of rhythm-related reward and emotion (RRRE) system

There are both external and internal rhythmic events that are relevant to an organism (Vitaterna et al., 2001; Rutter et al., 2002). The coordination of internal rhythm to an external rhythm is called entrainment (Merker et al., 2009; McAuley, 2010; Phillips-Silver et al., 2010) or sensorimotor synchronization (SMS) especially when the internal rhythm is movement (Repp and Su, 2013). Phillips-Silver et al. (2010) suggested there are various entrainments that build upon pre-existing adaptations which enable organisms to perceive, produce and synchronize with rhythmic stimuli.

**Figure 2:**
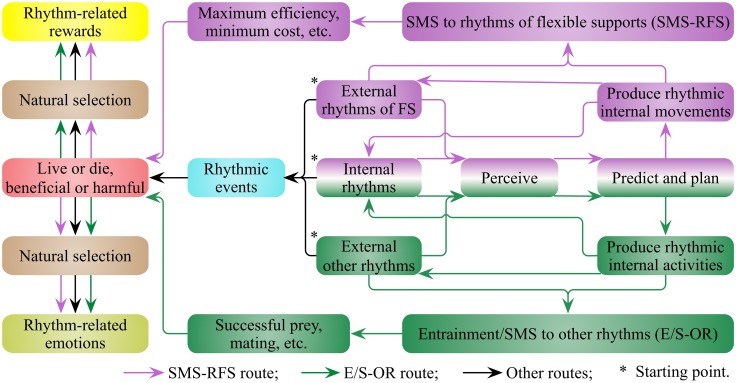
**The evolutionary routes of rhythm-related reward and emotion (RRRE) system**. There are external and internal rhythms for organisms. Organisms perceive, predict, plan and produce rhythms to synchronize with external rhythms. I suggest that the sensorimotor synchronization of organisms to rhythms of flexible supports (SMS-RFS) and the entrainments/SMS to other rhythms (E/S-OR) are so vital for the survival and reproduction of organisms that they drive the generation of the RRRE system. Results induced by rhythmic events also contribute a lot to the generation of RRRE system. FS, flexible supports.

I suggest that living environments such as water, air and trees, are rich in rhythmic movements produced through natural forces (wind and tide, etc.) and biological forces (animal activities). Both arboreal and aquatic animals have adapted in order to thrive in the flexible supports in which they live (Thorpe et al., 2009; Shepard et al., 2013). For example, killer whales and their prey, sea lions, both have distinguished swimming skills to deal with the fluctuant ocean environment for successful predation and escape, respectively (Vila et al., 2008). Animal movements also influence and are influenced by the surrounding flexible supports, which add additional forces to the animal locomotor system (Thorpe et al., 2009). Animals have to perceive and predict external and internal movements to plan and produce the next movement (Figure 2). I suggest that both types of movements of animals and those of the flexible supports are rhythms rich that can fit and interact with each other, as well as a lock and key. I define this as the sensorimotor synchronization to rhythms of flexible supports (SMS-RFS, Figure 2). Since the minimum cost of transport (COT_min_) is critical for survival (Shepard et al., 2013), orangutans do use tree sway to reduce the energetic cost of gap crossing (Thorpe et al., 2007), and it is less costly for northern gannets to fly with the wind than against it (Amelineau et al., 2014). I propose the evolutionary values of SMS-RFS are energy-saving, time-saving and efficiency of locomotion, which are vital to fierce competition for survival and reproduction.

Since the SMS-RFS and the entrainments/SMS to other rhythms (E/S-OR) are critical for survival and reproduction of organisms, I propose that they drive the generation of a RRRE system (Figure 2). The reward evoked by rhythm is probably not secondary, but primary, as rewards evoked by food and sex (Sescousse et al., 2013). Rhythmic events in nature also connect with various phenomena and lead to corresponding results, contributing to the generation and activation of the RRRE system. These rhythmic events lead to both beneficial and harmful results, arousing corresponding positive and negative everyday emotions, respectively. Under natural selection, the connections between rhythmic events and emotions are inherently established. Some rhythmic events arouse positive emotions and encourage animals close to it; on the contrary, some arouse negative emotions and drive animals to keep away from it. The RRRE system enables animals to appreciate rhythmic events, to perceive, produce and synchronize with rhythmic events, as well as to adopt advantageous and avoid disadvantageous rhythmic events.

## The biological origins and social evolution of pro-music, pro-dance, and pro-speech in nonhuman animals

**Figure 3:**
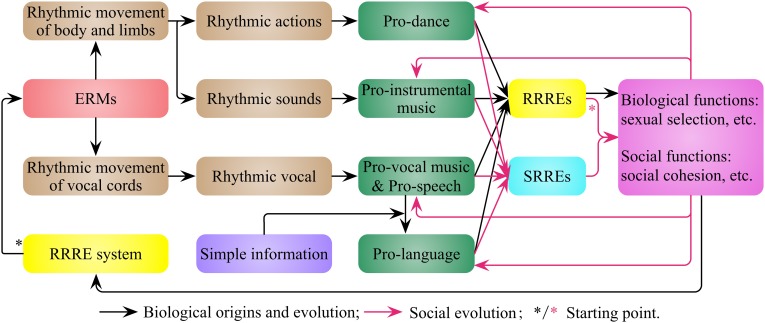
**The biological origins and social evolution of pro-music, pro-dance, and pro-speech in nonhuman animals**. The rhythm-related reward and emotion (RRRE) system (Figure 2) makes animals produce entertainment rhythmic movements (ERMs) and leads to pro-music, pro-dance, pro-speech and pro-language. The first type of rewards and emotions (rhythm-related rewards and emotions, RRREs) evoked by them have biological and social functions. These functions are the results of their biological origins, as well as the driving force for their evolution. These functions also enable the development of a second type of rewards and emotions (society-related rewards and emotions, SRREs).

I suggest that the RRRE system (Figure 2) enables animals to enjoy, search and produce rhythmic events, and drives the generation of entertainment rhythmic movements (ERMs), which are characterized by rhythm, aesthetics and fluency, and likely involve the refinements of everyday movements. Most of the ERMs probably are the refinements of everyday movements that have evolutionary value, and some ERMs may be driven by the RRRE system independently and have no obvious evolutionary value originally. The ERMs of the body and limbs is pro-dance, such as the dance of bird of paradise and crane (Mandoki, 2014), and the non-vocal rhythmic sound generated in this process is pro-instrumental music (Figure 3), such as the sound made by palm cockatoo's drumming (Wood, 1984; Gray et al., 2001). The rhythmic vocal sound generated by the rhythmic movement of vocal cords, such as bird and whale songs (Payne, 2000; Gray et al., 2001), is both pro-vocal music and pro-speech. Pro-language is the vocal sound that includes simple information. Therefore rhythmic vocal is often a combination of pro-vocal music, pro-speech and pro-language for animals, and the boundary among them is obscure.

The first type of rewards and emotions (rhythm-related rewards and emotions, RRREs) evoked by pro-music, pro-dance, pro-speech, and pro-language have biological functions (e.g., sexual selection) and social functions (e.g., fostering the social bonding). These biological and social roles are largely defined by their biological origins, and provide a driving force for their continued biological and social evolution. These functions also enable the development of a second type of rewards and emotions, which I name society-related rewards and emotions (SRREs, Figure 3).

## The biological origins and social evolution of music, dance, and language in human

**Figure 4:**
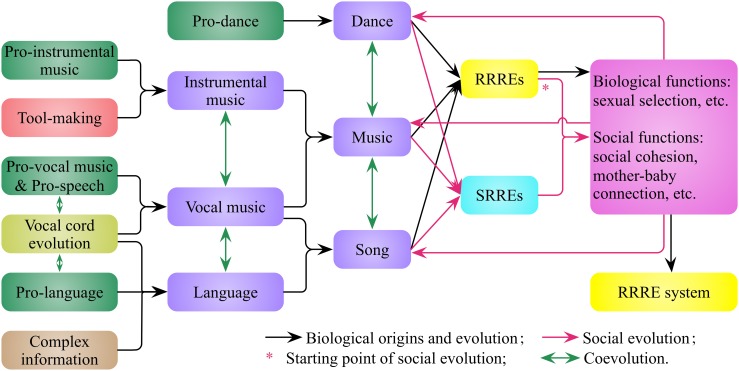
**The biological origins and social evolution of music, dance, and language in humans**. Tool making promotes the origins of instrumental music, vocal cord evolution promotes the origins of vocal music and language, and there are coevolutions among music, dance, and language. The rhythm-related rewards and emotions (RRREs) evoked by music and dance give rise to their biological and social functions, which in turn promote the biological and social evolution of music and dance, and enable the development of society-related rewards and emotions (SRREs) of music and dance. The neural circuits of RRREs and SRREs develop in species formation and personal growth, with congenital and acquired characteristics, respectively.

I propose that human beings inherit and develop the RRRE system from their aquatic and arboreal ancestors. On one hand, the coevolution of pro-vocal music, pro-speech, pro-language, and vocal cords leads to vocal music and language (Figure 4). Language is a vocal tool derived from pro-speech and evolved from pro-language for the purpose of complex information communication. On the other hand, tool making leads to instrumental music, such as the bone and ivory flutes from Hohle Fels and Vogelherd in Germany (Conard et al., 2009), and the bone flutes of Neanderthal (Gray et al., 2001). A melody represents two things, one is rhythmic audio event, the other is rhythmic movement interpreted by the Doppler effect (Figure 1), and both of them trigger the human RRRE system to evoke rewards and emotions.

The first type of rewards and emotions—RRREs—evoked by music and dance are beneficial for both individuals and populations. These rewards and emotions lead to biological functions, such as sexual selection (Miller, 2000), and social functions, such as fostering the mother-baby connection (Dissanayake, 2000; Trehub, 2003) and social cohesion (Brown, 2000a; Freeman, 2000; Mithen, 2007). These functions in turn promote the biological and social evolution of music and dance, also enable music and dance to evoke a second type of rewards and emotions—SRREs (Figure 4). I suggest that the neural circuits of RRREs and SRREs develop both congenital and acquired characteristics during species formation and personal growth, respectively. On one hand, from a biological perspective, the genetic basis of human musicality has undergone macroevolution and microevolution in the development of RRREs and SRREs, respectively. On the other hand, from a sociological perspective, the types and applications of music have undergone microevolution and macroevolution in the development of RRREs and SRREs, respectively. Therefore, music, dance, and speech are the combination of nature (biological origins) and culture (social evolution), and can induce both RRREs and SRREs.

**Figure 5:**
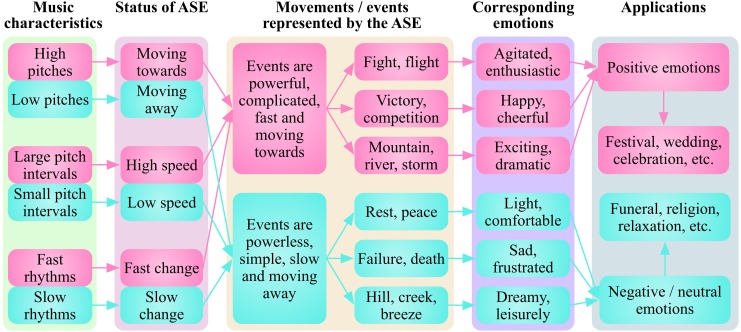
**Different music characteristics represent different rhythmic movements/events and induce corresponding emotions**. Generally, music with a high pitch, large pitch range and fast tempo induces happiness, excitement or fear, maybe because these characteristics represent fast and powerful movements/events such as fight, flight, competition or weather storms. In contrast, music with a low pitch, narrow pitch range and slow rhythm can induce sadness or peace, perhaps due to these characteristics represent slow and powerless movements/events such as weakness, wound, failure or a gentle breeze. ASE, abstract sounding events.

I suggest that the biological origins and social evolution of music endow the rewards and emotions evoked by music with species-specific and individual-specific traits. I propose that different characteristics in melodies corresponding to rhythmic movements, represent corresponding rhythmic events, and induce corresponding emotions that can be applied in corresponding social activities (Figure 5). Generally, music with a high pitch, large pitch range and fast tempo could induce happiness, excitement or fear (Gabrielsson and Juslin, 2003; Hunter and Schellenberg, 2010; Juslin and Sloboda, 2011), maybe because those characteristics represent fast and powerful movements/events such as fight, flight, competition, or weather storms. On the contrast, music with a low pitch, narrow pitch range and slow rhythm could induce sadness or peace (Gabrielsson and Juslin, 2003; Hunter and Schellenberg, 2010; Juslin and Sloboda, 2011), perhaps because these characteristics represent slow and powerless movements/events such as weakness, wound, failure, or a gentle breeze. This means that some common music characteristics may induce corresponding emotions through the RRRE system across human culture, which is consistent with the fact that basic emotions (happiness, sadness, and fear) in Western music can be recognized by the native African population (Fritz et al., 2009). However, even the same music may induce individual specific emotions (Brattico and Jacobsen, 2009; Mas-Herrero et al., 2013), with the development of SRREs depending mainly on the cultural, as well as individual experiences.

## Discussion

Music exists ubiquitously across human history and human culture (D'errico et al., 2003; Conard et al., 2009). What's still under debate is which selection pressures are responsible for the origins and evolution of music and musical emotions. Many reasonable theories, such as sexual selection (Darwin, 1871; Miller, 2000), mother-baby connection (Dissanayake, 2000; Trehub, 2003) and social cohesion (Brown, 2000a; Freeman, 2000; Mithen, 2007) have been previously considered as the selection pressures for music evolution. However, I suggest the primary selection pressure for human musicality is identical with the selection pressure for the RRRE system—the adaptation of organisms to ubiquitously and variously internal and external rhythmic movements and events. Sexual selection and those social functions (e.g., mother-baby connection and social cohesion) are results of music's biological origins, as well as the driving force for the evolution of music and the development of SRREs. That is to say, sexual selection and social cohesion are secondary selection pressures for the evolution of music.

Previous studies show that music can evoke rewards and emotions (Blood et al., 1999; Blood and Zatorre, 2001; Menon and Levitin, 2005; Koelsch et al., 2006; Koelsch, 2014). This hypothesis suggests the biological basis for music and dance is the RRRE system, whose selection pressure is the adaptation of organisms to ubiquitously external and internal rhythmic movements and events. As the synchronization of an organism to rhythmic events is even more pivotal to survival than food and sex to some extent, the reward evoked by rhythmic events is probably a primary reward, as with those evoked by food and sex (Sescousse et al., 2013). This may be the reason that music can trigger the mesolimbic reward system that is similarly triggered by food, sex and drugs (Blood and Zatorre, 2001; Zatorre and Salimpoor, 2013).

Previous studies indicate that body movement plays an important role in musical rhythm perception (Phillips-Silver and Trainor, 2005), also show music and movement shared a dynamic structure that supported universal expressions of emotion (Sievers et al., 2013). Here I propose certain pitch interval in music represents invariant movement pattern by the interpretation of Doppler effect. This hypothesis integrates music and dance in a common form—rhythmic movements. This may be able to explain the close relationships between music and movement. This hypothesis also provides a probable explanation for the transposition invariance of melodies in music perception, the reason may be that a series of invariant pitch intervals represent an invariant movement pattern.

Previous studies also indicate that music and speech may have evolved from a common ancestor (Brown, 2000b; Besson et al., 2011; Brandt et al., 2012). Here I suggest that speech and music are both derived from rhythmic vocalizations where the rhythmic vocal is often both pro-music and pro-speech, with the boundary between them obscure. Accordingly, I expect that more overlapping neural circuits for both music and speech should be existed in animals and children than human adults. Therefore, I suggest that vocal sounds used as signatures in some parrots (Berg et al., 2012), dolphins (King and Janik, 2013) and human tribe (Ammann et al., 2013) are both music and language to a degree.

I also suggest that SMS (one of the major characteristics of the RRRE system) is a necessary precondition for vocal mimicking. This view predicts that the ability to synchronize with an auditory rhythm likely exists in, but not confined to, vocal mimicking species. This is consistent with previous studies that SMS with music exists not only in multiple vocal mimicking species, such as humans (Repp, 2005) and parrots (Patel et al., 2009; Schachner et al., 2009), but also in non-vocal mimicking species, such as chimpanzees (Hattori et al., 2013) and sea lions (Cook et al., 2013).

This hypothesis suggests RRRE system shares both universal and species-specific characteristics. On one hand, the RRRE system is universal to both human and other animal species, especially those species living in flexible environments. That is consistent with the fact that almost all of the singing species such as canary, humpback whale, and white-handed gibbon are living in water, air or trees (Marler, 2000; Payne, 2000; Gray et al., 2001; Hauser and McDermott, 2003). It may be also the reason why whale song and bird song are similar in structure to human song and speech (Gray et al., 2001; Bolhuis et al., 2010). Furthermore, recent findings indicate that most songs of the hermit thrush make use of the same mathematical principles that underlie many human musical scales (Doolittle et al., 2014), and that chimpanzees prefer African and Indian music over silence (Mingle et al., 2014), both demonstrating common characteristics between humans and animals. Contrastingly, according to the proposed hypothesis, different species have differences between their RRRE systems, including differing sensorimotor organs. Even between chimpanzee and humans, there are differences in speech anatomy (Morley, 2003). It means that animals do not have to enjoy human music, but may enjoy it.

This hypothesis indicates that human musicality is not an all-or-none ability, but a comprehensive talent combining many sensorimotor abilities. More specifically, a sound musical talent in humans requires the entire RRRE system, as well as the sensorimotor systems for pitch, rhythm, and motion. Any deficit in these systems could lead to deficiency in a corresponding musical ability, but not musical abilities as a whole. For example, beat deaf amusia (Phillips-Silver et al., 2011; Palmer et al., 2014), pitch deaf amusia (Phillips-Silver et al., 2013), and specific musical anhedonia (Mas-Herrero et al., 2014) exhibit some normal musical perception capacities, despite having deficits in auditory beat perception, pitch cognition and musical pleasure extraction, respectively. In contrast, since music has close relations with the sensorimotor and emotional systems, if there is a deficit in those systems, music activity may play an alternative role to help recovery by facilitating the use and development of common neural circuits shared by music, movement, and emotion. This is consistent with previous studies in which music has the ability to cure patients who have movement or mental diseases (Altenmüller et al., 2009; Koelsch, 2014). For example, music can reduce pain and increase functional mobility in fibromyalgia (Garza-Villarreal et al., 2014).

Overall this hypothesis provides a probable selection pressure and outline for the evolution of music, dance and speech, and also reasonably explains most music phenomena investigated previously. I hope it shall enlighten future research on music, dance, and speech. Future experiments could allow for a clearer neurological definition of the RRRE system and SRRE circuits. The relationships between these two systems and the three phenomena in question (music, dance, and speech) should also be explored. Since optogenetics is a powerful technology that allows the fast and specific control of neural activities in brain of freely moving animals (Deisseroth, 2011), it has been applied in vocal learning research of songbirds successfully (Roberts et al., 2012; Roberts and Mooney, 2013). The optogenetics stimulation of rewarding regions efficiently guides the learning of sensory discrimination in mouse (Liu et al., 2014). Previous studies also indicate that music do effects physiology and psychology in mouse (Chikahisa et al., 2006; Angeluccia et al., 2007; Li et al., 2010; Uchiyama et al., 2012). Therefore I suggest that optogenetics can be used to establish animal models (mouse and/or bird) for music cognition on the behavioral, neural and genetic levels.

### Conflict of interest statement

The author declares that the research was conducted in the absence of any commercial or financial relationships that could be construed as a potential conflict of interest.
